# Top Three Strategies of ss(+)RNA Plant Viruses: Great Opportunists and Ecosystem Tuners with a Small Genome

**DOI:** 10.3390/v13112304

**Published:** 2021-11-18

**Authors:** Volodymyr V. Oberemok, Yelizaveta V. Puzanova, Anatoly V. Kubyshkin, Rina Kamenetsky-Goldstein

**Affiliations:** 1 Molecular Genetics and Biotechnologies Lab, V.I. Vernadsky Crimean Federal University, Simferopol 295007, Russia; genepcr@mail.ru; 2 Laboratory of Entomology and Phytopathology, Nikitsky Botanical Garden, National Scientific Centre, Russian Academy of Sciences, Yalta 298648, Russia; 3 Department of General and Clinical Pathophysiology, V.I. Vernadsky Crimean Federal University, Simferopol 295006, Russia; kubyshkin_av@mail.ru; 4 Institute of Plant Sciences, Agricultural Research Organization, The Volcani Center, Rishon LeZion 7505101, Israel; vhrkamen@volcani.agri.gov.il

**Keywords:** ss(+)RNA plant viruses, symbiosis, adaptation, RNA-dependent RNA polymerase, natural selection, DNA insecticides

## Abstract

ss(+)RNA viruses represent the dominant group of plant viruses. They owe their evolutionary superiority to the large number of mutations that occur during replication, courtesy of RNA-dependent RNA polymerase. Natural selection rewards successful viral subtypes, whose effective tuning of the ecosystem regulates the interactions between its participants. Thus, ss(+)RNA viruses act as shuttles for the functionally important genes of the participants in symbiotic relationships within the ecosystem, of which the most common ecological triad is “plant–virus–insect”. Due to their short life cycle and large number of offspring, RNA viruses act as skillful tuners of the ecosystem, which benefits both viruses and the system as a whole. A fundamental understanding of this aspect of the role played by viruses in the ecosystem makes it possible to apply this knowledge to the creation of DNA insecticides. In fact, since the genes that viruses are involved in transferring are functionally important for both insects and plants, silencing these genes (for example, in insects) can be used to regulate the pest population. RNA viruses are increasingly treated not as micropathogens but as necessary regulators of ecosystem balance.

## 1. Introduction

Viruses are highly adaptable to their environments because a huge number of genotypes emerge from the replication process, owing to the peculiarities of RNA-dependent RNA polymerase. During difficult times, the unique viral life cycle allows the survival of sometimes only a few host cells under conditions with access to minimal resources. Mutations play an important role in saving viruses since a large number of viral particles die during the struggle of the fittest genotypes to survive. Plant viruses seek refuge in the host cell, achieving a certain balance between reproduction and the consumption of its resources, and finding evolutionarily advantageous mechanisms that allow symbiotic coexistence. Among plant viruses, the ss(+)RNA group has been rewarded by evolution and has flourished, blessed, on the one hand, with a small genome, and, on the other, with the ability to constantly change its genome and to withstand various environmental factors. In addition, when passing through the “bottleneck”, they give rise to a new subtype or group of subtypes adapted to competitive environmental conditions. Viruses have moved beyond mere participation in the “micropathogen–host” relationship to become an important link for other ecosystem participants [1]. It is natural that, in many ways, their life cycles are interwoven with those of another group of organisms experiencing evolutionary abundance—insects [2]. The triangle “plant–virus–insect” best describes the life cycle of plant ss(+)RNA viruses, where the plant is the place of reproduction, and the insect is the distributor, and sometimes the place of reproduction, as well. The virus needs to find a balance between the two organisms to ensure maximum fitness. In this article, we will try to expand this triangle into a square by including humans, whose understanding of how the relationships in the ecological triangle “plant–virus–insect” function will help in the practical application of this knowledge, both for selective insect control and for rethinking the fight against viral diseases in plants.

## 2. Micro Boy Scouts: Always Ready to Translate

ss(+)RNA viruses belong to the most ancient and dominant group of plant viruses, currently numbering more than 430 species from 6 families [3]. The key adaptative strategy used by ss(+)RNA viruses exploits the possibility of rapid translation after entering the cell since the genome of ss(+)RNA viruses is essentially a matrix RNA, ready for instant translation [4] using the biosynthetic apparatus of the host cells. It is noteworthy that the structural features of plant tissues and cells, namely, the presence of plasmodesmata, allow the virus to efficiently and quickly spread its RNA between plant cells without reproducing new virions [5]. The genome of ss(+)RNA viruses is a polycistronic RNA containing information for several proteins, usually no more than 10. The main proteins of ss(+)RNA viruses are RNA-dependent RNA polymerase, structural proteins of the capsid and outer membrane, and helicases and genes whose products contribute to spreading the virus. The genome of ss(+)RNA viruses has a capped 5′-end containing N-formylmethionine, which promotes the initiation of protein biosynthesis, and a polyadenylated 3′-end, which helps evade destruction by intracellular nucleases [6]. These two structural features are related to the mRNA of the host cells and help the virus avoid degradation and/or an immune response from the plant. The absence of a nuclear phase in the life cycle of ss(+)RNA viruses accelerates the time from the ingress of viral particles to the assembly of new virions in the cell [7]. The compactness of the genome is ensured by shifting the reading frame, whereby several proteins are encoded at once at one site [8]. During evolution in close proximity to their hosts, ss(+)RNA viruses gain the ability to encode proteins and regulators of various functions in the minimal space of the RNA sequence [9]. When necessary, a plant RNA virus hijacks endocytic proteins to establish its infection in plants [10].

dsRNA, ss(−)RNA, ssDNA, and dsDNA viruses have various intermediate stages associated with the formation of mRNA that require a substantial amount of time and energy to set up and begin productive reproduction in the host cell. However, ss(+)RNA viruses have the shortest genome (genus *Ourmiavirus*: 0.97–2.8 kb) among the RNA-containing viruses. The largest genome among ss(+)RNA viruses belongs to viruses in the Closteroviridae family (13–19.3 kb). A short genome allows the elegant reproduction of viral quasi-individuals that differ from the “parent” by one–two mutations at the lowest cost for the host cell [11]. In addition, the genomes of ss(+)RNA viruses lack the informational redundancy of double-stranded genomes [12]. Thus, ss(+)RNA viruses of plants, in a sense, can be considered pure information which requires minimum effort and energy to reproduce. Once ensconced in favorable conditions in the host cell, ss(+)RNA plant viruses trigger the effective replication of their genomes.

## 3. Never-Ending Treasures of Mutations

For ss(+)RNA viruses, the issue of adaptation to the environment is particularly acute since it is not able to actively move in search of a host. Therefore, when a micropathogen enters a cell, it must present all its best qualities to be able to replicate. To sort out all the best options for interaction with the host cell, these options must first be created. The key role in this is played by RNA-dependent RNA polymerase, which lacks a corrective ability. The error rate of RNA-dependent RNA polymerase is estimated at 10^−4^, whereas in DNA viruses, this indicator is 10^−6^–10^−7^ [13]. Genome size negatively correlates with mutation rate [14].

Viruses have an r-development strategy, and only a small number of virions will reach a new target in the form of a new host individual after leaving the old one. They need a lot of offspring, a small genome, a high degree of overlap, and a small number of alternative pathways in the case of mutation, indicating a weak protection of the virus genome [15]. The most variable part of the virus genome is the one responsible for interaction with the cell membrane [16]; the most conservative is the RNA-dependent RNA polymerase gene and some structural protein genes that make up the capsid [17]. After studying a large body of literature, we concluded that the frequency of mutations decreases for proteins located deeper inside the mature viral particle, whereas for proteins that come into contact with membrane receptors and help penetrate the host cell, this frequency is higher. This is consistent with the concept of natural selection and microevolution, aimed at maximizing the fitness of viruses for their hosts. Despite the generally accepted notion that most mutations cause damage to their carrier, and that the frequency of positive mutations is negligible, viruses maintain their mutation rate close to the threshold of lethal mutagenesis, occupying a unique ecological niche. Walking this tightrope has a safety net: viruses can remain outside the host cell for a long time without dying, waiting for their lucky break. During this time, certain host cells may also change so that some genotypes of the virus will be maximally adapted to the host cells, which was not the case before. In this sense, ss(+)RNA viruses can delay adaptability when no new genotypes are created, holding it in reserve for when environmental conditions change, some of which will be favorable for this “micro boy scout” who is always ready for translation.

## 4. Tiny Ecosystem Tuners

Due to the economic importance of plant viruses, most of the information accumulated about them to date is related to the pathogenic viruses of agricultural plants. Because humans rely on agriculture for survival, this leads to the erroneous opinion that any virus causes damage to plants. However, closer examination of the structure of natural ecosystems shows that viruses, for the most part, do not harm their hosts; in fact, it is safe to say that a significant number of viruses enter into mutually beneficial relationships with their plant hosts. Viruses are very common in wild plants, and they tend to be asymptomatic [18]. To a certain extent, the virus does not benefit from the death of the host cell; moreover, the replication of the ss(+)RNA virus genome depends on a wide range of factors in the host itself. This makes adjustments for the assembly of the virus replication complex [19]. There are examples of the labile influence of viruses on their hosts, when the relationship between them jumps from mutualistic to antagonistic and vice versa. For example, under normal conditions for a plant, the virus manifests itself as a pathogen, but once environmental conditions change to threaten the existence of the plant, the virus helps its host cope with abiotic and even biotic stress [20]. A number of viruses confer drought resistance on their hosts, and some help plants cope with cold stress [21]. Metabolite profiling analysis showed an increase in several osmoprotectants and antioxidants in brome mosaic virus-infected rice and cucumber mosaic virus-infected beet plants before and after drought stress. There are viruses that negatively affect host phytophages [22]. For example, white clover mosaic virus infection can decrease the attractiveness of white clover plants for fungus gnat females due to β-caryophyllene. These aspects of viral infection clearly show how viruses, existing in the same territory as plants, insects, and microorganisms, defend their borders and become more involved in the ecosystem, adjusting and complementing it. Viruses adapt precisely and exceptionally well to their “guardians”, given their nutrient status [23].

**Figure 1:**
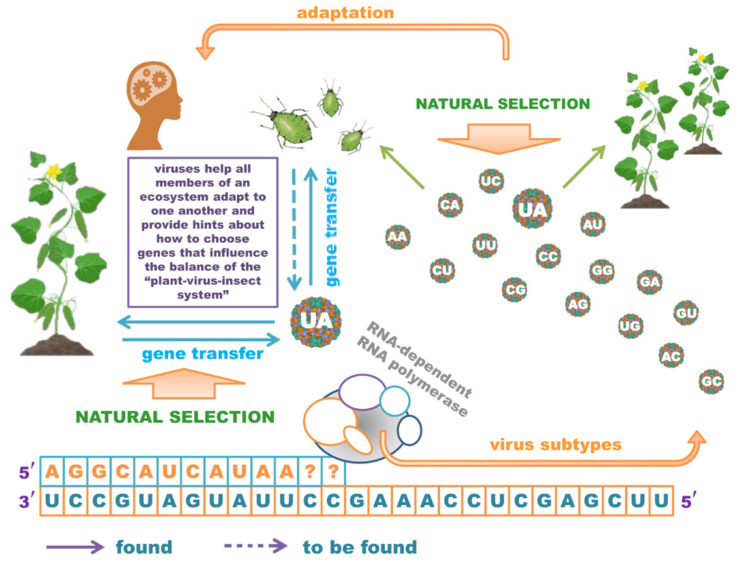
Tuning of ecosystems by ss(+)RNA viruses.

In our opinion, in modern terms, a virus should not be considered an organism but instead a “program” that facilitates the process of transmitting genetic information. Viruses help increase genetic diversity and are discernible in phenomena such as crossing over, mutation, and independent chromosome divergence. When viruses multiply very intensively, it means that the participants of the ecosystem are not sufficiently adapted to each other. A large number of scientific papers agree that it is viruses, along with microorganisms, that carry out gene transfer between different types of organisms in the ecosystem. For many ss(+)RNA viruses, the possibility of exchanging genome fragments in the form of endogenous viral elements (EVE) in insects [24] and plants [25], or in genes hijacked from plants [18], has been demonstrated in a variety of ecosystem participants. In our opinion, the hijacking of insect genes by viruses is very likely to be detected in the very near future (Figure 1). This is especially true for plant viruses that can multiply in the tissues of insect carriers. Above all, this will apply to monophagous insects, for whom adaptation to the plant is vital.

This ability to move back and forth among the members of an ecosystem indicates the possibility that viruses are active participants in the genetic transformation of the genoplast of the community [26].

In a sense, we see nucleic acids as a key regulator of ecosystem life. If we view viruses as “coated nucleic acids”, then they represent the simplest manifestation of life that can affect the ecosystem’s balance. For example, outbreaks of viral diseases in agroecosystems may not just be a consequence of the low biodiversity of the system [27], but rather the result of viruses actively working to restore balance and increase biodiversity. A promising offshoot of this concept is the possibility of using gene sequences transferred from one organism to another, since, as a rule, these functionally important genes ensure the maximum adaptation of organisms to each other and therefore play an important role in the cell. Blocking these genes can be used to create ways to control insect pests.

Oberemok’s research group has been developing DNA insecticides since 2008 [28,29] and sees ss(+)RNA viruses as a convenient platform for creating highly effective DNA insecticides against insect pests from the suborder Sternorrhyncha of the order Hemiptera, as it was shown for 28S ribosomal genes [30,31]. We plan to further use homologous EVEs found in the “plant–virus–insect pest” triangle to create DNA insecticides, as well. Another promising approach using the information contained in the genome of viruses is the use of DNA insecticides together with viral preparations. Our data show the possibility of the joint use of very short antisense fragments of the anti-apoptotic genes of DNA-containing baculoviruses and preparations of *Lymantria dispar* multicapsid nuclear polyhedrosis virus (LdMNPV) to combat the reproduction and further spread of the gypsy moth, thereby confirming this principle in the fight against insect pests. Baculoviruses are ubiquitous in the environment and are known to be an important regulator of insect populations. In the presence of LdMNPV infection, it is better to rely on alterations in the expression of functionally important virus genes (for example, inhibitor of apoptosis (IAP) genes) that could have an insecticidal effect on gypsy moths. Phylogenetic analysis of baculoviral IAP genes indicated their host origin [32,33], and some baculovirus IAPs bear a striking resemblance to the cellular IAPs carried by the host insects that they infect [34]. Our results indicate the possibility of using antisense oligonucleotide oligoRING (5′-CGACGTGGTGGCACGGCG-3′) of the LdMNPV IAP3 gene and LdMNPV preparations (one following the other) to control gypsy moths and to encourage this principle in plant protection against insect pests [35,36].

Insect pest control always involves disseminating a significant portion of the preparation used into the environment. Unmodified oligonucleotides seem to be the safest way to do this since cells contain ubiquitous nucleases that can neutralize them [30], embodying the idea of green insecticides. Thus, the “plant–virus–insect” triangle can be controlled by knowing which genes are important for its existence and dynamic development, and which are actively transferred between organisms with the participation of viruses, including ss(+)RNA viruses. By expanding the triangle to a square, i.e., by including humans, data concerning its functioning can be used to control agricultural systems.

## 5. Conclusions

In our opinion, ss(+)RNA plant viruses manifest themselves as great opportunists and ecosystem tuners. This is a consequence of their short life cycle, the peculiarities of the functioning of RNA-dependent RNA polymerase, and the minimum number of substances necessary for the reproduction of viral particles. They adapt easily to various environmental factors, which has allowed them to survive and flourish. At the same time, they act as programmers of the ecosystem by transferring functionally important genes, often using insects as a vehicle, to fine-tune the symbiotic relationships among its participants. For plants, as the main solar energy accumulators and producers, viruses are a necessary component of the ecosystem; by efficiently adjusting how the food network functions, they ensure the sustainable development of its participants in symbiotic relationships. The fundamental knowledge of the role played by RNA viruses in an ecosystem can be used to effectively and safely regulate the number of insect pests using the DNA insecticide approach.
